# Haploinsufficiency of *Dspp* Gene Causes Dentin Dysplasia Type II in Mice

**DOI:** 10.3389/fphys.2020.593626

**Published:** 2020-11-09

**Authors:** Ce Shi, Ning Ma, Wei Zhang, Jiapeng Ye, Haibo Shi, Danwei Xiang, Chunyue Wu, Lina Song, Ning Zhang, Qilin Liu

**Affiliations:** ^1^Department of Oral Pathology, School and Hospital of Stomatology, Jilin University, Changchun, China; ^2^Jilin Provincial Key Laboratory of Tooth Development and Bone Remodeling, School and Hospital of Jilin University, Changchun, China; ^3^Department of Rheumatology, First Hospital of Jilin University, Changchun, China; ^4^Department of Oral and Maxillofacial Surgery, School and Hospital of Jilin University, Changchun, China

**Keywords:** heterozygous, periodontium, dentin, *DSPP*, DD-II

## Abstract

Dentin dysplasia (DD) and dentinogenesis imperfecta (DGI) patients have abnormal structure, morphology, and function of dentin. DD-II, DGI-II, and DGI-III are caused by heterozygous mutations in the dentin sialophosphoprotein (*DSPP*) gene in humans. Evidences have shown that loss of function of DSPP in *Dspp* knockout mice leads to phenotypes similar to DGI-III, and that the abnormal dentinogenesis is associated with decreased levels of DSPP, indicating that DSPP haploinsufficiency may play a role in dentinogenesis. Thus, to testify the haploinsufficiency of *Dspp*, we used a *Dspp* heterozygous mouse model to observe the phenotypes in the teeth and the surrounding tissues. We found that *Dspp* heterozygous mice displayed dentin phenotypes similar to DD-II at the ages of 12 and 18 months, which was characterized by excessive attrition of the enamel at the occlusal surfaces, thicker floor dentin of the pulp chamber, decreased pulp volume, and compromised mineralization of the dentin. In addition, the periodontium was also affected, exhibiting apical proliferation of the junctional epithelium, decreased height and width of the alveolar bone, and infiltration of the inflammatory cells, leading to the destruction of the periodontium. Both the dental and periodontal phenotypes were age-dependent, which were more severe at 18 months old than those at 12 months old. Our report is the first to claim the haploinsufficiency of *Dspp* gene and a DD-II mouse model, which can be further used to study the molecular mechanisms of DD-II.

## Introduction

Dentin dysplasia (DD) and dentinogenesis imperfecta (DGI) are autosomal dominant genetic diseases characterized by abnormal formation of the dentin. DD has two subgroups, type I (DD-I) and type II (DD-II). The teeth in patients with DD-I have a normal clinical appearance; however, radiologically, the roots are pointy and short or even no roots. In patients with DD-II, the primary teeth are opalescent with obliterated pulp chambers. In contrast, the permanent teeth are normal in clinical appearance, but show pulpal calcification, including mild diffuse mineralization or frequent pulpal stones. Genetically, DD-I is found to be related to mutations in *SMOC2*, *SSUH2*, *VPS4B* genes (Chen et al., 2019), while DD-II is caused by mutations in the dentin sialophosphoprotein (*DSPP*) gene. For DGI, there are type I (DGI-I), II (DGI-II), and III (DGI-III). DGI-I occurs as part of osteogenesis imperfecta, in which bones are brittle and easily broken. DGI-II is the most common type of DGI, exhibiting an amber or opalescent dentin, short roots, and missing pulp chambers. DGI-III patients have opalescent primary and permanent teeth, with large pulp chambers which are contrary to DGI-II. DGI-I patients have mutations in *COL1A1* or *COL1A2*, while DGI-II and DGI-III patients have been identified with mutations in the *DSPP* gene. The above classification was proposed by Shields et al. (1973). However, some case reports described clinical manifestations that belonged to several of these diseases, making the diagnosis and classification difficult for practitioners. Thus, scholars proposed to reclassify the three diseases, DD-II, DGI-II, and DGI-III, caused by *DSPP* gene mutations, into DGI mild, moderate, and severe form, respectively (de La Dure-Molla et al., 2015).

To characterize the molecular events that regulate dentinogenesis during normal tooth development and in the conditions of DD and DGI, studies have been performed on animal models using *Dspp* knockout mice. As early as 2003, the *Dspp*-null mice was generated, which developed severe dental defects characterized by decreased mineralization of the dentin, increased width of the predentin and increased pulp volume, resembling DGI-III patients (Sreenath et al., 2003). We also found the DGI-III-like phenotypes in *Dspp* knockout mice at the ages of 3 and 6 months (Gibson et al., 2013a). Besides, we and others found that *Dspp* knockout mice also developed periodontal defects (Gibson et al., 2013b, 2014), as well as accelerated secretion and maturation of enamel (Verdelis et al., 2016). However, *Dspp* heterozygous mice did not show any abnormalities, and the authors suggested that haploinsufficiency did not play a role in these mice (Suzuki et al., 2009). Intriguingly, human genetic studies showed that heterozygous but not homozygous mutation of the *DSPP* gene was causative for DD and DGI patients, and most DD and DGI patients are inherited in an autosomal dominant fashion. Thus, it is likely that DSPP manifested haploinsufficiency.

To testify the haploinsufficiency of *Dspp* gene, it is necessary and essential to use *Dspp* heterozygous mice, which can further understand the function of *Dspp* and its products, and help to interpret the relationship between the mutations and the clinical manifestations. Therefore, in this study, *Dspp* heterozygous mice was employed and the phenotypes in the teeth and the periodontium was examined in detail.

## Materials and Methods

### Generation of *Dspp* Homozygous and Heterozygous Mice

*Dspp*^+/–^ mice was obtained by *Dspp*^–/–^ mice (C57BL/6 genetic background, generated in NIDCR) (Sreenath et al., 2003) crossbred with C57BL/6 wild-type mice. *Dspp*^+/–^ mice were then crossbred to generate wild-type (*Dspp*^+/+^, designated as WT hereafter), *Dspp* heterozygous (*Dspp*^+/–^, designated as HET hereafter), and *Dspp* homozygous knockout (*Dspp*^–/–^, designated as KO hereafter) mice. Adequate measures were taken to minimize pain or discomfort of the experimental animals. The animal study was reviewed and approved by the Animal Care and Use Committee at Jilin University (Norman Bethune College of Medicine 2018098).

### Micro-Computed Tomography (Micro-CT) Scanning and Pulp Volume Measurement

Mandibles from WT, HET, and KO mice at the ages of 12 and 18 months old (*n* = 5 for each group) were harvested and fixed in 4% paraformaldehyde (PFA). The mandibles were scanned using a micro-CT system (μCT50, Scanco Medical AG, Bassersdorf, Switzerland) with a 12-μm voxel size using the following parameters: 114 mA, 70 kVp, and exposure time of 300 ms.

To evaluate bone mass, the interradicular septum between the two roots of the mandibular first molars were chosen. Bone mineral density (BMD), tissue mineral density (TMD), bone volume fraction (BV/TV), trabecular number (Tb. N), trabecular thickness (Tb. Th), and trabecular separation (Tb. Sp) were analyzed. To identify the mineralization of dentin, TMD within the coronal dentin of the mandibular first molars were measured, which can be easily distinguished from the enamel by the density of the scanned images.

To measure the pulp volume of the mandibular first molars and incisors, Mimics 21.0 was utilized to complete the segmentation or virtual reconstruction (Ranjitkar et al., 2019). Mask volumes were quantitatively analyzed in mm^3^.

### Histology, Histochemistry, and Immunohistochemistry Staining

Mandibles were fixed in 4% PFA, decalcified with 10% EDTA, and embedded in paraffin. Sagittal sections were made at 4 μm, and stained for hematoxylin and eosin (HE) and tartrate-resistant acid phosphatase (TRAP). For TRAP staining, the slides were incubated with TRAP working solution for 60 min at 37°C, and then counterstained with hematoxylin. For the quantification of osteoclasts, the numbers of osteoclasts were divided by the length of the bone surface. Osteoclasts were defined as TRAP-positive with ≥3 nuclei.

For histology, histochemistry, and immunohistochemistry (IHC) staining, sections were incubated with rat monoclonal antibodies against DSP (1:500, anti-DSP-2C12.3) and BSP (1:400, anti-BSP-10D9.2) (from Dr. Chunlin Qin in Texas A&M University) for overnight at 4°C. For negative control, rat IgG was used instead of the primary antibodies. A mouse-on-mouse (MOM) kit and diaminobenzidine tetrachloride (DAB) were used. Methyl green was used for counterstaining.

### Statistical Analysis

Data were shown as the means ± standard deviations (SD) of triplicates. Data analyses were conducted with SPSS 19.0 and based on the normal distribution examination using the Kolmogorov–Smirnov test and homogeneity of variance evaluation using Levene’s test. For comparisons among three groups, data differences were assessed using one-way ANOVA and Tukey’s Honestly Significant Difference. For comparisons between two groups, unpaired student tests were used. Values of *P* < 0.05 were considered significant.

## Results

### *Dspp* Heterozygous Mice Resembled DD-II Radiographically

**FIGURE 1:**
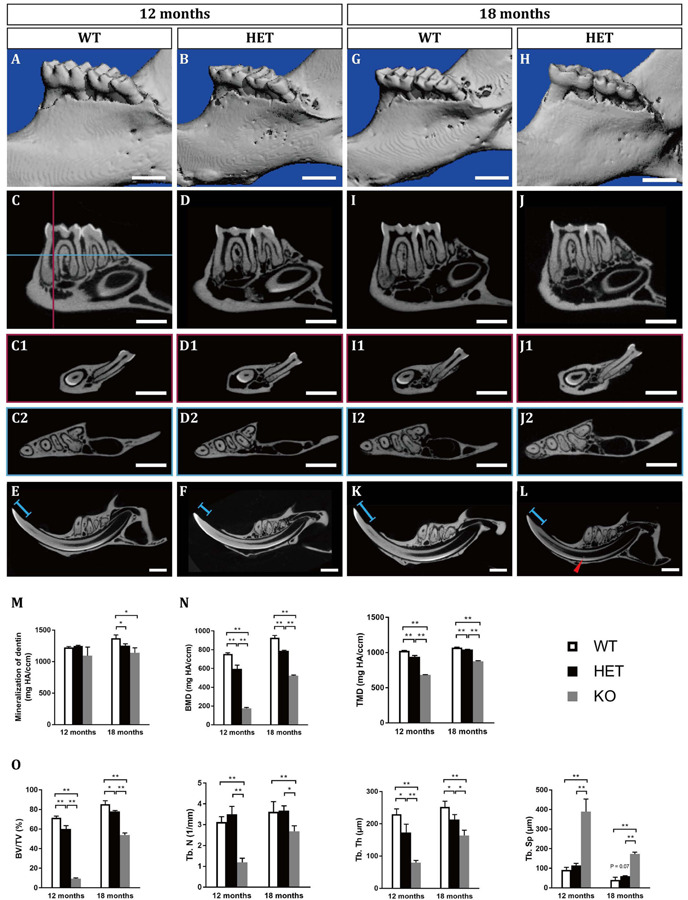
Tooth phenotypes in *Dspp* heterozygous mice at the ages of 12 months **(A–F)** and 18 months **(G–L)**, resembling dentin dysplasia type II (DD-II). **(A,B,G,H)** Reconstruction images of the micro-CT scanning in wild type (WT, *Dspp*^+/+^) and *Dspp* heterozygous (HET, *Dspp*^+/–^) mice. **(C,D,I,J)** Sagittal sections of the mandibles in WT and HET mice, showing the largest sections of the mandibular first molars and the mandibular second molars. **(C1,D1,I1,J1)** represented the coronal sections of the largest sections of the mesial roots of the mandibular first molars, as illustrated in **(C)** the vertical red line. **(C2,D2,I2,J2)** represented the horizontal sections of the mandibles, as illustrated in **(C)** the horizontal blue line which was approximately 0.3 mm below the alveolar ridge at the furcation of the mandibular first molars. **(E,F,K,L)** Sagittal sections of the mandibular incisors. Red arrowheads pointed to the pulpal stones in the mandibular incisors. Blue lines indicated the lengths between the incisures on the lingual sides of the mandibular incisors and the cusps. **(M)** The mineralization of dentin of the mandibular first molars. **(N,O)** Parameters related with interradicular septum between the two roots of the mandibular first molars. BMD, bone mineral density; TMD, tissue mineral density; BV/TV, bone volume fraction; Tb. N, trabecular number; Tb. Th, trabecular thickness; Tb. Sp, trabecular separation. For each group, *n* = 5. *P < 0.05; **P < 0.01. Scale bars = 1 mm.

**FIGURE 2:**
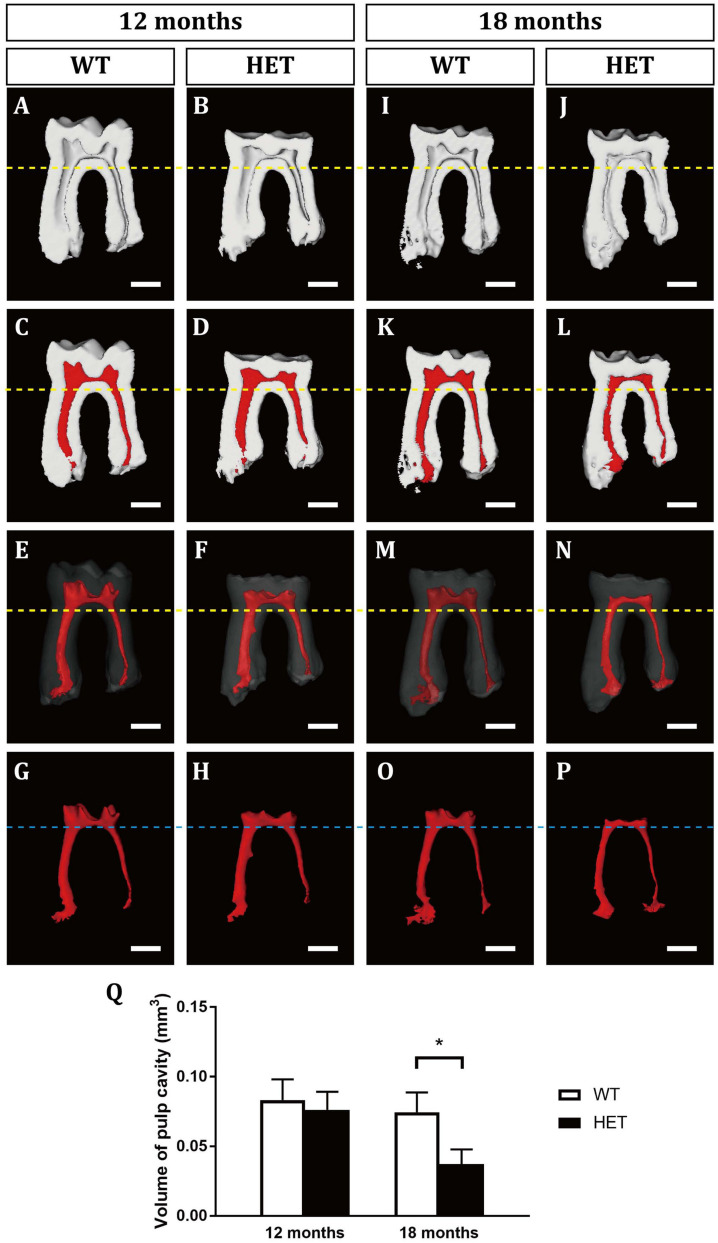
3D virtual reconstructions of the mandibular first molars in wild-type and *Dspp* heterozygous mice at the ages of 12 months **(A–H)** and 18 months **(I–P)**. **(A,B,I,J)** showed the sagittal sections of the mandibular first molars by micro-CT. **(C,D,K,L)** were images of the mandibular first molars with the pulp cavity in red. **(E,F,M,N)** showed the 3D images of the mandibular first molars with the enamel and dentine transparent and pulp cavity in red. **(G,H,O,P)** were the 3D morphologies of the pulp cavity. Yellow dotted lines marked the levels of the bifurcation of the mandibular first molars. Blue dotted line marked the levels of the floor of the pulp chambers of the mandibular first molars. **(Q)** The measurements of the pulp volumes. For each group, *n* = 5. *P < 0.05. Scale bars = 0.5 mm.

At the age of 12 months, the HET mice showed excessive attrition of the enamel at the occlusal surfaces, compared with the WT control (Figures 1A,B). Sagittal sections showed that the enamel of the occlusal surfaces of the mandibular first molars was almost gone in the HET mice, leaving a much thinner layer of dentin (Figures 1C,D). However, there was no difference in the pulp volume (Figure 2 and Supplementary Table 2) or the mineralization of dentin of the mandibular first molars between the WT and the HET group (Figures 1C,D,M and Supplementary Table 1). In contrast, the pulp volume of the incisors in the HET group showed a decreased tendency (*P* = 0.055) (Supplementary Figure 3). Moreover, obvious incisures (Figures 1E,F, ends of the blue lines) on the lingual sides of the mandibular incisors can be visualized in WT mice, indicating cementum dentin junction, while the incisures in HET mice were not obvious, along with shorter length between the incisures and the incisor cusps (Figures 1E,F, blue lines). These morphological changes in the mandibular incisors confirmed that the teeth in the HET mice were ill-mineralized and more prone to attrition. Collectively, these results demonstrated that knockout of one allele of *Dspp* was sufficient to cause DD-II in mice, indicating haploinsufficiency of *Dspp* gene. With regard to the alveolar bones, there were less bone mass and more pores in the HET group, compared with the WT group (Figures 1C,D,N,O and Supplementary Table 1).

At the age of 18 months, the HET group showed similar but more severe phenotypes compared with those at 12 months, including the excessive attrition of the enamel at the occlusal surfaces (Figures 1G–J), thinner roof dentin but thicker floor dentin of the pulp chambers leading to decreased pulp volume (Figures 1I,J, 2 and Supplementary Table 2), decreased mineralization of dentin (Figure 1M and Supplementary Table 1), less bone mass in the alveolar bones (Figures 1I,J,N,O and Supplementary Table 1). For the mandibular incisors, the HET group exhibited thinner dentin and enamel with decreased mineralization, less obvious incisures on the lingual sides and shorter lengths between the incisures and the incisor cusps (blue lines in Figures 1K,L). Besides, the HET mice also had pulpal stones in the mandibular incisors (Figure 1L, red arrowhead). Contrary to the mandibular first molars, the pulp volume of the incisors were significantly increased in the HET group, compared with the WT controls (Supplementary Figure 3). We also found that some of the HET mice show more severe phenotypes, including obliterated pulp cavities and multiple pulpal stones (Supplementary Figures 2A–C). Collectively, these results demonstrated that the phenotypes in HET mice were similar to DD-II patients and were age-dependent.

We also included *Dspp* homozygous knockout mice for micro-CT scanning (Supplementary Figure 1). These KO mice bore more severe DGI-III-like phenotypes, exhibiting decreased dentin thickness and increased pulp volumes. The KO phenotypes were also age-dependent (Figures 1M–O, Supplementary Figure 1, and Supplementary Table 1).

### *Dspp* Heterozygous Mice Exhibited Malformed Dentin and Decreased Volume of the Pulp Chambers

**FIGURE 3:**
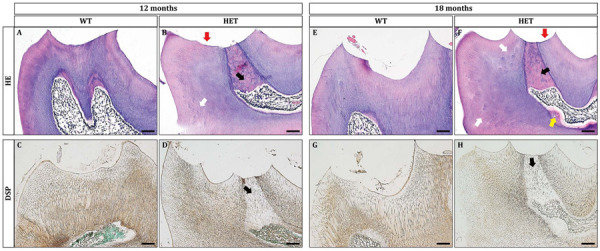
*Dspp* heterozygous mice exhibited dentin malformation at the ages of 12 months and 18 months. **(A,B,E,F)** HE staining showed that *Dspp* heterozygous mice **(B,F)** had aberrant reparative dentin (black arrows) beneath the attrited cusps (red arrows) or on the wall of the pulp chamber (yellow arrow). White arrows in **(B,F)** pointed to the hypomineralization in *Dspp* heterozygous mice. **(C,D,G,H)** DSP immunohistochemical staining. Sagittal sections of the crowns of the mandibular first molars were shown. The DSP positive signals in the dentin of the *Dspp* heterozygous mice **(D,H)** was relatively weaker than that in the WT mice **(C,G)**. Reparative dentin showed much more weaker DSP than normal dentin [black arrows in **(D)** and **(H)**]. For each group, *n* = 3. Scale bars = 50 μm.

Then, we examined the histological changes of the mandibular first molars. At 12 months old, HE staining showed that the cusps in the HET group were excessively abrased (Figure 3B, red arrow). Correspondingly, there was excessive amount of reparative dentin formation in the pulp opposing the attrited cusps (Figure 3B, black arrow). On the contrary, there was little attrition or no tertiary dentin formation in the WT group (Figure 3A). Noteworthy, there were spots of dentin globules beneath the mantle dentin in the HET group (Figure 3B, white arrow), suggesting the less mineralization. The results of DSP immunohistochemical stainings showed that the positive signals of DSP in the HET group were much weaker, compared with the WT group (Figures 3C,D). The DSP positive signals in the reparative dentin (black arrow) were much weaker than the circumpulpal dentin in the HET group (Figure 3D).

The phenotypes were also age-dependent. At the age of 18 months, the HET group manifested excessive dentin attrition (Figures 3E,F, red arrow), excessive formation of reparative dentin (Figures 3E,F, black arrow), more dentin globules (Figures 3E,F, white arrows), smaller pulp chamber partly due to dentin formation at the floor of the pulp chamber (Figures 3E,F, yellow arrow), significantly decreased DSP immuno-reactive signals within both the reparative dentin (Figures 3G,H, black arrow) and the circumpulpal dentin (Figures 3G,H). The formation of reparative dentin at the wall of the pulp chamber (Figures 3E,F, yellow arrow) suggested that the reparative dentin was not, or at least not fully, attrition induced. The much weaker DSP signals within the reparative dentin indicated osteodentin rather than normally formed dentin. The decreased protein levels of DSP in the dentin of HET mice suggested that ablation of one allele of *Dspp* gene significantly reduced its protein products, confirming the haploinsufficiency. The products of *Dspp*, as the main non-collagenous extracellular matrices of dentin, regulate the mineralization of dentin. Thus, it is reasonable to observe that the mineralization of dentin was compromised in HET mice, leading to excessive attrition. In addition, the more severe phenotypes in some of the HET mice showed much thicker tertiary dentin both at the attrited cusps (Supplementary Figures 2E,F) and at the floor of the pulp chamber and within the root canals (Supplementary Figure 2D).

### Periodontium Was Affected in *Dspp* Heterozygous Mice

**FIGURE 4:**
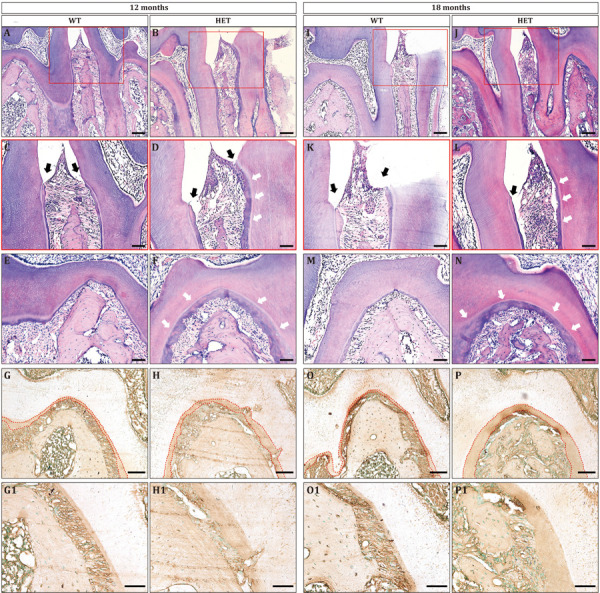
Periodontitis and cementum hyperplasia could be found in *Dspp* heterozygous mice at the ages of 12 months **(A–H)** and 18 months **(I–P)**. **(A,B,I,J)** Sagittal sections of the interdental septum between the mandibular first molars and the mandibular second molars. Scale bar = 100 μm. **(C,D,K,L)** Enlarged images of the red boxes in **(A,B,I,J)**, respectively. Black arrows indicated the level of epithelial attachment and the bottom of the gingival sulcus. White arrows pointed to the thickened cementum. Scale bar = 50 μm. **(E,F,M,N)** Furcation areas of the mandibular first molars. Scale bar = 50 μm. **(G,H,O,P)** Immunohistochemical staining for BSP at the furcation areas, with red dotted lines illustrating the area of the cementum. **(G1,H1,O1,P1)** are the high magnification images of **G,H,O,P**, respectively. For each group, *n* = 3. Scale bars = 50 μm.

To gain more insights into the haploinsufficiency effect of *Dspp* gene, we then observe the histology of periodontium. At the age of 12 months, interdental papillae between the mandibular first and second molars could be seen in both WT and HET group (Figures 4A,B). However, the junctional epithelium proliferated apically in the HET group, attaching to the cementum (Figure 4D, black arrows); on the contrary, the junctional epithelium in the WT group was attached to the enamel (Figure 4C, black arrows). The height and width of the alveolar bone beneath the interdental papillae were decreased in the HET group, leading to increased width of the periodontal ligament, compared with the WT group (Figures 4C,D). The cementum was significantly thicker in the HET group than that in the WT group, both on the surface of the roots (Figures 4C,D, white arrows) and within the furcation areas (Figures 4E,F). The thickness of the cementum was confirmed by BSP immunostaining (Figures 4G,H), since BSP was specifically expressed in the extracellular matrix of the cementum.

The phenotypes of periodontitis were much more severe at the age of 18 months, including apically proliferative junctional epithelium (Figures 4I–L), decreased height and width of the alveolar bone (Figures 4I,J), and increased thickness of cementum (Figures 4K–P). In addition, there were large amounts of inflammatory cells in the periodontium of the HET group (Figures 4J,L), leading to the destruction of the epithelium of the interdental papillae and the collagen fibers of the periodontal ligament. Collectively, *Dspp* heterozygous mice developed severe periodontitis as aging.

**FIGURE 5:**
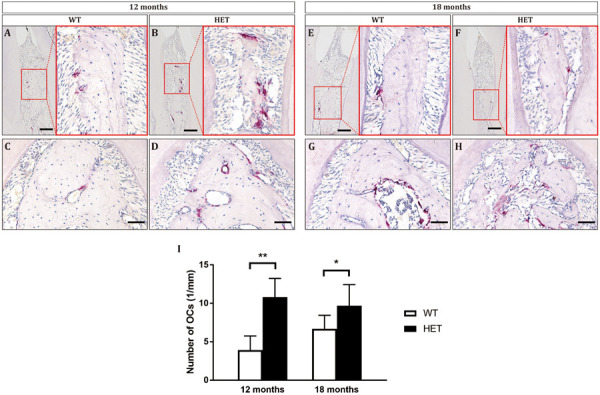
Osteoclasts were over-activated in *Dspp* heterozygous mice, leading to decreased alveolar bone mass and height at the ages of 12 months **(A–D)** and 18 months **(E–H)**. Tartrate-resistant acid phosphatase (TRAP) staining showed the sagittal sections of the interdental septum between the mandibular first molars and the mandibular second molars **(A,B,E,F)**, as well as sagittal sections of the furcation areas of the mandibular first molars **(C,D,G,H)**. Scale bar in **A,B,E,F** = 50 μm. Scale bar in **C,D,G,H** = 20 μm. **(I)** The numbers of osteoclasts (TRAP positive with no less than three nuclei) on the surface of alveolar bone were counted. For each group, *n* = 3. *P < 0.05; **P < 0.01.

To assess the level of osteoclastic bone resorption, TRAP staining was performed. There were more matured osteoclasts on the surface of the alveolar bone in the HET group, compared with the WT group (Figure 5 and Supplementary Table 3). Besides, within the interdental septum, the osteoclasts in the WT group were located in the mesial surface (Figures 5A,E), whereas the osteoclasts in the HET group were located in both the mesial and distal surface (Figures 5B,F), leading to significantly decreased height and width of the interdental septum. These results indicated that the decreased alveolar bone mass was due to over-activated osteoclastic bone resorption in the HET group.

## Discussion

In this study, to verify the haploinsufficiency of *Dspp* gene on dentinogenesis, we investigated the phenotypes of teeth in *Dspp* heterozygous mice at the ages of 12 and 18 months. We found that *Dspp* heterozygous mice developed DD-II-like dental phenotypes, including normal color but excessive attrition of the occlusal surface, thicker floor dentin and smaller volumes of the pulp chambers, pulpal stones, and hypomineralization of dentin. In addition, periodontium in *Dspp* heterozygous mice was disrupted, including apical proliferation of the junctional epithelium, destruction of the alveolar bone, and infiltration of the inflammatory cells. To the best of our knowledge, our report was the first to claim the haploinsufficiency of *Dspp* gene in mice and a DD-II-like mouse model.

Our findings were contrary to the publications, which addressed no dental phenotypes in the *Dspp* heterozygous mice (Suzuki et al., 2009; Verdelis et al., 2016; Zhang et al., 2018). However, the heterozygous mice used in their studies were younger than 6 months old. While, in our current study, we observed the phenotypes in mice of 12 months and 18 months old, and the phenotypes were age-dependent. Thus, it is reasonable that in *Dspp* heterozygous mice, there were no arresting phenotypes when the mice were younger than 6 months old. Nevertheless, we still do not know when the heterozygous mice began to show phenotypes.

The DD-II-like phenotypes in our current mice were milder and detected later, compared with the corresponding DD-II patients. This could be explained by the integrated theories of haploinsufficiency and dominant negative effects. In our *Dspp*^+/–^ mice, the DSPP products were reduced to 50% compared with the wild type, since exons 2–5 were ablated (Sreenath et al., 2003). The DD-II-like phenotypes in our mouse model demonstrated that *Dspp* gene is characterized by haploinsufficiency. What is more, our results together with the publications indicated that the levels of the DSPP products played an essential role in dentinogenesis (Sreenath et al., 2003), since *Dspp*^–/–^ mice exhibited the most severe DGI-III-like phenotypes whereas *Dspp*^+/–^ mice manifested the mildest DD-II-like phenotypes. On the other hand, the dominant negative effects of DSPP is clearly demonstrated by the fact that in odontoblasts of DD and DGI, the mutant DSPP proteins entrap wild type DSPP through Ca^2+^-binding domains, and are retained within rough endoplasmic reticulum (rER) (von Marschall et al., 2012; Liang et al., 2019). It is supposed that the effect of DD-II associated mutation in capturing the wild type DSPP within rER is lower than that of DGI (von Marschall et al., 2012), resulting in the mildest phenotypes in DD-II among the three diseases. Thus, in comparison with our mouse model, DD-II patients are more severe, due to entrapment of the mutant and normal DSPP protein within rER although there are 50% normal *Dspp* gene and its products, resulting in less than 50% secretion of normal DSPP and the abnormal biology of odontoblasts.

Interestingly, Lee et al. (2008) have reported that one splice junction mutation (IVS2-6T > G) in DD-II patients produced more than half of the normal amount of DSPP protein, due to a percentage of the pre-mRNA from the diseased allele would splice normally and generate wild-type mRNA. They also speculated that a small amount of (about 25%) reduction of the normal DSPP protein resulted in the phenotype of DD type II, while haploinsufficiency of *DSPP* in humans resulted in the more severe forms of inherited dentin defects, DGI-II and DGI-III, which differ from the observation of our *Dspp*^+/–^ mouse model. In fact, in patients of DD-II, DGI-II, or III, it is difficult to predict the effects of these mutations on *Dspp* transcript, as well as the quantity of normal DSPP, since the mRNA of mutated *Dspp* allele may have different fate, either being degraded or generating mutant DSPP protein, due to diversiform mutation patterns (Shyu et al., 2008). It is worth noting that the so called 50% reduction in the amount of DSPP product in *Dspp*^+/–^ mice is only theoretical, while real-time polymerase chain reaction (RT-PCR) or protein quantitative assay are warranted, since feedback mechanism of *Dspp* haploinsufficiency may occur, especially in odontoblasts. Therefore, to uncover the genotype-phenotype correlationship, the cause to induce the interesting phenotypes, both in transgenic/gene knockout animal models and diseased human kindred, need to be further studied.

Although the mice used in our current study exhibited DD-II-like phenotypes in molars, the pulp volumes of the incisors were higher along with much thinner dentin at the age of 18 months (Supplementary Figure 3). In physical conditions, as opposed to molars that stop growing after occlusion, mouse incisors continuously growing to compensate for the masticatory abrasion throughout life (Verdelis et al., 2016). Therefore, we conjectured that the contraries between molars and incisors were due to that the dentin of the HET mice were more prone to be attrited, leading to more rapid compensatory growth of the incisors beyond dentin deposition (Burn-Murdoch, 1999). This result also suggested a distinction between molars and incisors, in terms of development, signaling regulation, and function, as evidence by a plethora of studies (Li et al., 2011; Laugel-Haushalter et al., 2013).

Dentin sialophosphoprotein was initially found to be specifically expressed by odontoblasts and transiently by pre-ameloblasts (D’Souza et al., 1992; Begue-Kirn et al., 1998), while it was later observed also in bone (Qin et al., 2002), periodontium (Baba et al., 2004) and other non-mineralized tissues (Ogbureke and Fisher, 2004, 2007;Sun et al., 2010; Prasad et al., 2011). Patients with DGI have compromised alveolar bone cells (Porntaveetus et al., 2019), suggesting that these patients may suffer from periodontal diseases. DGI-III animal model (*Dspp* null mice) developed severe periodontitis, also displayed impaired cranial bone development and long bone mineralization (Verdelis et al., 2008; Gibson et al., 2013b; Chen et al., 2015). Herein, our study demonstrated that *Dspp* heterozygous mice exhibited periodontal defects similar to periodontitis. Also, *Dspp* heterozygous mice manifested accelerated cementification and obvious osteoporosis within the bifurcation areas. Overall, due to the extensive expressions of DSPP in multiple tissues (D’Souza et al., 1992, 1997; Begue-Kirn et al., 1998; Qin et al., 2002, 2003; Baba et al., 2004), as well as the facts that phenotypes within the teeth, periodontium, and bone were observed in *Dspp* mutant mice (Verdelis et al., 2008; Gibson et al., 2013a,b, 2014), the concept that DD-II, DGI-II, and DGI-III are diseases only restricted to the teeth should be renewed. Thus, we should pay attention not only to the teeth of the patients with DD and DGI, but also to the state of periodontal health and bone homeostasis.

## Conclusion

In summary, our study demonstrated that *Dspp* heterozygous mice showed dentin and periodontal phenotype similar to DD-II and periodontitis in an age-dependent manner. We also presented for the first time the evidence of haploinsufficiency of *Dspp* gene and a DD-II mouse model, which can be used to further study the underlying pathogenesis and molecular mechanisms, under the premise of no genetic mouse model harboring the corresponding mutations in the DSPP gene of DD-II patients.

## Data Availability Statement

The raw data supporting the conclusions of this article will be made available by the authors, without undue reservation.

## Ethics Statement

The animal study was reviewed and approved by the Animal Care and Use Committee at Jilin University (Norman Bethune College of Medicine 2018098).

## Author Contributions

CS and NM contributed to data acquisition, analysis and interpretation, drafted the manuscript, and critically revised the manuscript. WZ contributed to data analysis and interpretation and critically revised the manuscript. JY, HS, DX, CW, LS, and NZ contributed to data acquisition and analysis and drafted the manuscript. QL contributed to conception, design and data interpretation, and drafted and critically revised the manuscript. All authors gave their final approval and agreed to be accountable for all aspects of the work.

## Conflict of Interest

The authors declare that the research was conducted in the absence of any commercial or financial relationships that could be construed as a potential conflict of interest.
