# Supplementary material for: Haploinsufficiency of Dspp Gene Causes Dentin Dysplasia Type II in Mice
Source: Front Physiol. 2020 Nov 9;11:593626. doi: 10.3389/fphys.2020.593626 (PMC7680915; doi:10.3389/fphys.2020.593626)
Supplement: Supplementary file 1 [file Data_Sheet_1.docx]

Supplementary Material

# Supplementary Figures

**Supplementary Figure 1.** *Dspp* homozygous knockout (*Dspp* ^-/-^, KO) showed more severe phenotypes than *Dspp* heterozygous knockout mice at the ages of 12 months **(A-C)** and 18 months **(D-F)** by micro-CT. **(A and D),** Reconstruction images of the micro-CT scannings. **(B and E),** Sagittal sections of the mandibles, showing the largest sections of the mandibular first molars and the mandibular second molars. **(B1 and E1)** represented the coronal sections of the largest sections of the mesial roots of the mandibular first molars. **(B2 and E2)** represented the horizontal sections of the mandibles, which was

approximately 0.3 mm below the alveolar ridge at the furcation of the mandibular first molars. **(C and F)**, Sagittal sections of the mandibular incisors. For each group, n = 4. Scale bar = 1 mm.


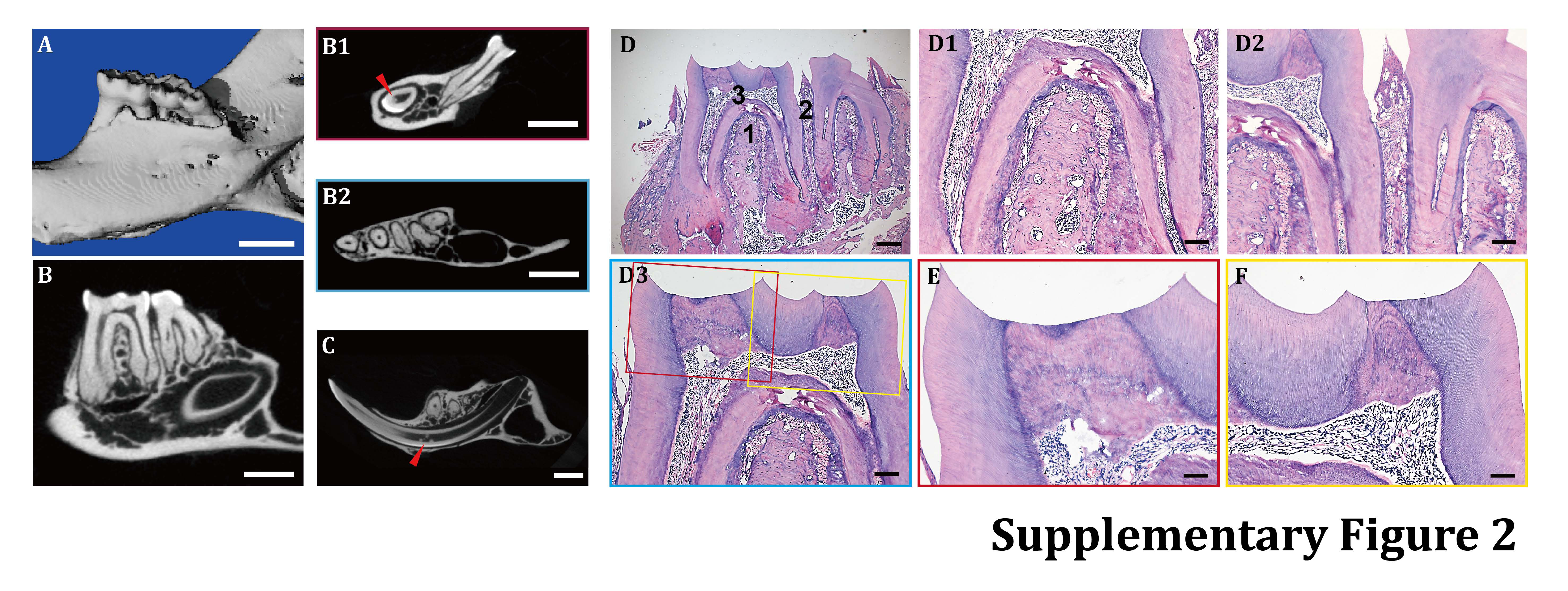


**Supplementary Figure 2.** *Dspp* heterozygous knockout mice exhibited severe phenotypes at the age of 18 months. **(A),** Reconstruction images of the micro-CT scanning. **(B),** Sagittal sections of the mandibles, showing the largest sections of the mandibular first molars and the mandibular second molars. **(B1 and B2)** represented the coronal and the horizontal sections as illustrated in Figure 1 legend, respectively. **(C)**, Sagittal sections of the mandibular incisors. Red arrowhead pointed to the multiple pulpal stones in the mandibular incisors. Scale bars in A-C = 1 mm. **(D-F),** HE staining of the mandibular first molars. **(D1, D2, and D3)** were the enlarged images of the corresponding numbers in **(D)**. **(E and F)** were the enlarged images of the corresponding boxes in **(D3)**. Scale bar in **(D)** = 200 μm. Scale bars in **(D1-D3)** = 100 μm. Scale bars in **(E and F)** = 50 μm.


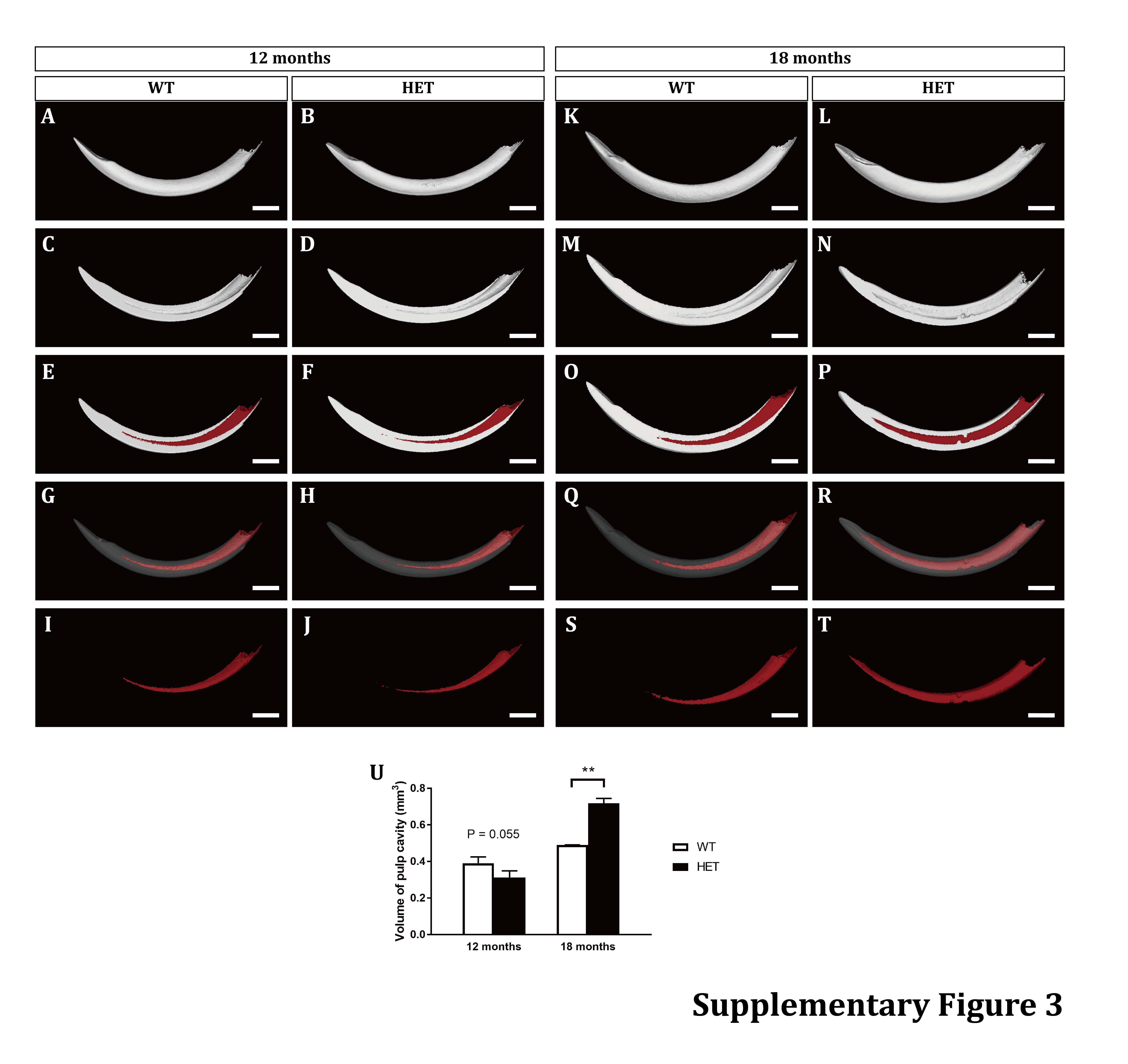


**Supplementary Figure 3.** 3D virtual reconstructions of the mandibular incisors in wild type and *Dspp* heterozygous knockout mice at the ages of 12 months **(A-J)** and 18 months **(K-T)**. **(A, B, K and L)** show the 3D reconstructions of the mandibular incisors. **(C, D, M and N)** are sagittal sections of the mandibular first molars by micro-CT. **(E, F, O and P)** are images of the mandibular incisors with the pulp cavity in red. **(G, H, Q and R)** show the 3D images of the mandibular first molars with the enamel and dentine transparent and pulp cavity in red. **(I, J, S and T)** are the 3D morphologies of the pulp cavity. **(U),** The measurements of the pulp volumes. For each group, n = 5. **: P < 0.01. Scale bar = 1 mm.

# Supplementary Tables

**Supplementary Table 1. Micro-Computed Tomography Analysis**

|  | **12 months** | **18 months** |
| --- | --- | --- |
| **Mineralization of dentin (mg HA/ccm)** |  |  |
| WT | 1223.57 ± 17.06 | 1254.77 ± 28.11 |
| HET | 1250.80 ± 8.79 | 1371.49 ± 50.85 ^a*^ |
| KO | 1096.75 ± 134.38 | 1139.84 ± 79.16 ^a*^ |
| **Bone mineral density (mg HA/ccm)** |  |  |
| WT | 754.46 ± 13.26 | 927.83 ± 23.26 |
| HET | 595.47 ± 38.99 ^a**^ | 787.24 ± 6.64 ^a**^ |
| KO | 174.92 ± 8.79 ^a**, b**^ | 522.50 ± 7.37 ^a**, b**^ |
| **Tissue mineral density (mg HA/ccm)** |  |  |
| WT | 1023.60 ± 5.89 | 1069.28 ± 6.54 |
| HET | 595.47 ± 38.99 ^a**^ | 1042.23 ± 4.75 ^a**^ |
| KO | 174.92 ± 8.79 ^a**, b**^ | 874.86 ± 9.18 ^a**, b**^ |
| **Bone volume fraction (%)** |  |  |
| WT | 71.51 ± 1.71 | 85.57 ± 3.29 |
| HET | 60.98 ± 3.34 ^a**^ | 78.13 ± 0.87 ^a*^ |
| KO | 9.31 ± 0.75 ^a**, b**^ | 53.90 ± 2.15 ^a**, b**^ |
| **Trabecular number (1/mm)** |  |  |
| WT | 3.12 ± 0.26 | 3.62 ± 0.47 |
| HET | 3.52 ± 0.35 | 3.67 ± 0.25 |
| KO | 1.18 ± 0.20 ^a**, b**^ | 2.68 ± 0.24 ^a**, b*^ |
| **Trabecular thickness (μm)** |  |  |
| WT | 229.66 ± 16.45 | 252.16 ± 16.86 |
| HET | 173.34 ± 25.52 ^a*^ | 213.34 ± 15.06 ^a*^ |
| KO | 79.58 ± 6.30 ^a**, b**^ | 163.96 ± 16.25 ^a**, b*^ |
| **Trabecular Separation (μm)** |  |  |
| WT | 91.94 ± 12.17 | 39.74 ± 13.85 |
| HET | 114.32 ± 10.02 | 59.92 ± 2.01 |
| KO | 397.92 ± 32.74 ^a**, b**^ | 172.66 ± 10.27 ^a**, b**^ |

n = 5. Values are mean ± SD.

a, statistically different from WT (α = 0.05).

b, statistically different from HET (α = 0.05).

*, P < 0.05; **, P < 0.01.

**Supplementary Table 2. Volumes of pulp cavity of mandibular first molars (mm^3^)**

|  | **12 months** | **18 months** |
| --- | --- | --- |
| WT | 0.084 ± 0.014 | 0.074 ± 0.014 |
| HET | 0.076 ± 0.011 | 0.038 ± 0.010 ^a*^ |

n = 5. Values are mean ± SD.

a, statistically different from WT (α = 0.05).

*, P < 0.05.

**Supplementary Table 3. Numbers of osteoclasts (1/mm)**

|  | **12 months** | **18 months** |
| --- | --- | --- |
| WT | 3.92 ± 1.61 | 6.67 ± 1.52 |
| HET | 10.87 ± 2.56 ^a^ | 8.56 ± 3.66 ^a^ |

n = 3. Values are mean ± SD.

a, statistically different from WT (α = 0.05).

*, P < 0.05.
